# The association between baseline persistent pain and weight change in patients attending a specialist weight management service

**DOI:** 10.1371/journal.pone.0179227

**Published:** 2017-06-12

**Authors:** Cormac G. Ryan, Arutchelvam Vijayaraman, Victoria Denny, Alison Ogier, Louisa Ells, Shaun Wellburn, Lesley Cooper, Denis J. Martin, Greg Atkinson

**Affiliations:** 1 Health and Social Care Institute, Teesside University, Middlesbrough, United Kingdom; 2 Specialist Weight Management Service, South Tees Hospitals NHS Foundation Trust, NHS, Middlesbrough, United Kingdom; Tokai Daigaku, JAPAN

## Abstract

**Objective:**

To quantify the influence of baseline pain levels on weight change at one-year follow-up in patients attending a National Health Service specialist weight management programme.

**Methods:**

We compared one-year follow-up weight (body mass) change between patient sub-groups of none-to-mild, moderate, and severe pain at baseline. A mean sub-group difference in weight change of ≥5kg was considered clinically relevant.

**Results:**

Of the 141 complete cases, n = 43 (30.5%) reported none-to-mild pain, n = 44 (31.2%) reported moderate pain, and n = 54 (38.3%) reported severe pain. Covariate-adjusted mean weight loss (95%CI) was similar for those with none-to-mild (8.1kg (4.2 to 12.0kg)) and moderate pain (8.3kg (4.9 to 11.7kg). The mean weight loss of 3.0kg (-0.4 to 6.4kg) for the severe pain group was 5.1kg (-0.6 to 10.7, p = 0.08) lower than the none-to-mild pain group and 5.3kg (0.4 to 10.2kg, p = 0.03) lower than the moderate pain group.

**Conclusions:**

Patients with severe pain upon entry to a specialist weight management service in England achieve a smaller mean weight loss at one-year follow-up than those with none-to-moderate pain. The magnitude of the difference in mean weight loss was clinically relevant, highlighting the importance of addressing severe persistent pain in obese patients undertaking weight management programmes.

## Introduction

Obesity is a major public health issue affecting one in four adults in England [1]. As such, strategies to enhance the effectiveness of weight loss services are of national importance [2] and it is essential that the weight management services that are in place are appropriate and fit for purpose. Chronic pain affects 13% of people in the UK [3]. There is a substantial body of evidence demonstrating a link between obesity and chronic pain [4–15]. A dose response relationship exists, with the prevalence of pain increasing with progressively higher BMI [14]. Whilst, the full extent of this relationship has yet to be explored, it is likely to be bi-directional and may be underpinned by a range of mechanical, physiological, psychosocial, and behavioural mechanisms [16,17].

Clinically, pain has been implicated as an important barrier to weight loss [18] and the management of obesity-related conditions such as diabetes [19]. Chronic pain can have negative effects on an individual’s diet via mechanisms such as hedonic (non-hunger related) eating [11]. Additionally, pain can impede physical activity [20] and activities of daily living [21], thus hindering weight loss. Chronic pain may also adversely affect an individual’s mood, which can have negative implications for weight loss via dysregulated stress systems or unhealthy lifestyles [11,22,23]. However, few studies have directly investigated the impact of persistent pain on weight loss.

Wachholtz et al [21] found that 83% of patients on a 4-week intensive weight loss program in the USA reported pain. Patient sex, influenced the pain and obesity relationship, with joint pain identified as a predictor of weight loss in women but not men. In a recent secondary analysis of an RCT investigating a weight loss intervention for patients with co-existing pain and overweight/obesity 80% reported moderate or severe pain [24]. Those with severe pain reported significantly less weight loss (-0.1%) compared to those with moderate (-1.9%) or no pain (-2.1%) [24]. These findings support the work of Wachholtz et al [21] and demonstrate that pain may be a considerable barrier to weight loss. However, the participants in this US study were veterans, 85% of whom were male. Thus, it is unclear if these findings would generalise to patients undergoing weight management interventions within the National Health Service (NHS) in England where up to 88% of patients are female [25] and women receive 75% of bariatric surgery [1]. Thus there is a need to specifically investigate the potential effect of pain on weight loss in this context. The aim of this study was to investigate the effect of persistent pain on weight loss in individuals receiving NHS specialist weight management services.

## Methods

### Participants

This is an analysis of an NHS clinical dataset of patients who attended a specialist weight management service in the North East of England from February 2013 to November 2014. To be referred to the specialist weight management service patients were required to meet the following admissions criteria of having a BMI of ≥40 or a BMI ≥35 with a significant co-morbidity such as diabetes or hypertension. Furthermore, patients were required to be registered with a local GP; aged ≥16years; with an ability to take charge of their dietary intake; assessed as “ready to change”; and have had previous attempts at weight loss either in primary care including community weight management programmes, exercise programmes or anti-obesity medication for a minimum of 6 months. Their GP needed to have completed a recent metabolic and endocrine assessment and could show that the patient’s underlying endocrine diagnosis was stable and any secondary causes of obesity excluded. Patients were excluded from referral to the specialist weight management service if they did not meet the admission criteria above, or if they had a suspected or diagnosed malignancy, were pregnant, or requiring post-bariatric care (unless previously known to the service). From the pre-existing patient database, to be included in this study, participants needed to have provided baseline and one-year outcome data. Ethical approval for this study was obtained from The School of Health and Social Care Research Ethics and Governance Committee at Teesside University (Reference number 074/14) and the Wales 7 National Research Ethics Committee (Reference number 14/WA/1050). The IRB waived the need for individual participant consent for medical records to be used in this study, and data was accessed anonymously.

### Intervention

The specialist weight management service provides a multidisciplinary, biopsychosocial approach for morbidly obese patients.

Patient treatment programmes consist of three main phases. The timing of these phases varied from patient to patient. In phase 1, patents initially receive a multidisciplinary team (MDT) assessment including consultation with a Dietician, Physiotherapist, Psychologist, Metabolic Physician/Endocrinologist, GP with a specialist interest in obesity management, and an individual care plan is generated. The individual care plan includes: an exercise and physical activity plan; outcomes expected for the individual; target weight; behavioural goals; modification of eating patterns; goals relating to lifestyle factors; changes in behaviour relating to triggers and barriers; food and activity diaries; tools and educational materials. In phase 2, patients move into group services and treatment according to their specific needs and care plan. During this phase a weekly drop-in and telephone support service is provided. Interactions with these elements of the service are recorded and shared with the patient’s Care Manager. In phase 3, patients are discharged from the service with details of the patient’s outcomes and an ongoing care plan sent to their GP; signposting to support groups; community weight management services and exercise groups for further weight loss and/or weight maintenance support.

### Outcome measures

Whilst outcome measures within the specialist weight management service are collected at regular intervals this study includes only the baseline and one-year post baseline data. The primary outcome measure was weight (body mass) loss, which was measured using a weighing scales (SECA 645 hand rail scale). Height was also recorded using a Leicester Height Measure (Mark 2) so that BMI could be calculated.

Pain was measured using the Short-form 36 (SF36) bodily pain subscale [26,27]. The scale includes two questions 1) *how much bodily pain have you had during the past four weeks*? and 2) *during the past four weeks*, *how much did pain interfere with your normal work (including work both outside the home and housework)*? The first question is rated on a 6-point Likert scale ranging from *none* to *very severe*, whilst the second question is rated on a 5-point Likert scale ranging from *not at all* to *extremely*. The raw score is then converted as a simple algebraic sum into a 0–100% scale value with higher scores representing higher pain levels [27]. The SF36 bodily pain scale is widely used and has demonstrated good levels of validity and reliability as a measure of pain [27–29].

The following additional participant characteristics were collected: sex, age, socioeconomic status and depression levels. Socioeconomic status was assessed using the Lower layer Super Output Area (LSOA) which is derived from the patient’s postcode. The LSOA was used to assign each patient an index of multiple deprivation, which was categorised into deciles with 1 being least affluent and 10 being most affluent. Depression levels were measured using the depression subscale of the Hospital Anxiety and Depression scale (HADs) [30].

### Statistical analysis

Individuals were categorised into none-to-mild, moderate, and severe pain sub-groups according to their baseline pain scores. The cut-off points used in this analysis were <50% mild pain, 50–69.99% moderate pain, and 70–100% severe pain [31]. A general linear model was used with weight loss (kg) as the dependent variable and pain subgroup as the independent variable (fixed effect). This model was covariate-adjusted for any differences in baseline weight, age, sex, socioeconomic status, and depression levels between sub-groups. Covariate-adjusted subgroup mean differences in weight loss and associated 95% confidence intervals (95%CI) were estimated for our primary comparisons.

A sub-group difference in mean weight change was considered clinically relevant if it was ≥5kg. This was based upon the American Heart Association guidelines which state that reductions in weight of 2.5–5.5kg achieved through lifestyle interventions can reduce the risk of developing type 2 diabetes in overweight and obese individuals by 30–60%, while a reduction of 5-8kg can improve triglyceride levels and blood lipid profile [32].

## Results

Data were obtained for 167 participants who provided baseline and one-year follow-up data. Of these, 26 had missing data and were thus excluded from the analysis. The descriptive characteristics for those with complete data and those with missing data are shown in Table 1. There was no substantial difference for outcome or exposure variables between those with complete and incomplete data.

**Table 1:** Key characteristics for complete case and missing data groups.

	Complete n = 141	Missing n = 26
Age (yrs)	52.2 (11.9)	52.5 (14.6)
Sex		
Men	30%	31%
Women	70%	69%
Socioeconomic status (1–10)	3 (1–6)	2 (1–4.5)
Depression (0–21)	8.0 (4.4)	8.8 (4.2)
Height (m)	1.65 (0.09)	1.65 (0.11)
Weight (kg)	127.2 (23.0)	130.7 (25.1)
Weight change (kg)	6.2 (11.5)	7.5 (7.5)
Weight change (%)	-4.9	-5.7
≥5kg weight loss achieved	52%	52%
BMI (kg.m^-2^)	46.3 (7.2)	47.6 (8.4)
Pain (0–100%)	60.3 (26.9)	66.7 (24.3)

Data are mean (SD) unless stated

Median and IQR is presented for socioeconomic status

In the missing group column n = 26 for all variables except: socioeconomic status n = 14, depression n = 23, weight change kg and % n = 21, 5kg and 5% weight loss achieved n = 21, pain n = 16.

Of the 141 complete cases, over the one-year period 52% of patients lost ≥5kg, which is a greater proportion than that expected due to typical within-subjects variation in weight [33]. The adjusted mean weight loss for the pooled sample was 6.5kg (95% CI 4.6 to 8.4kg) equivalent to a loss of 5.1% of initial weight. The average pain levels at baseline were 60.3% (SD 26.9%). When broken down into the pain subgroups, n = 43 (30.5%) reported none-to-mild pain (of which n = 6 reported no pain), n = 44 (31.2%) reported moderate pain, and n = 54 (38.3%) reported severe pain.

**Fig 1:**
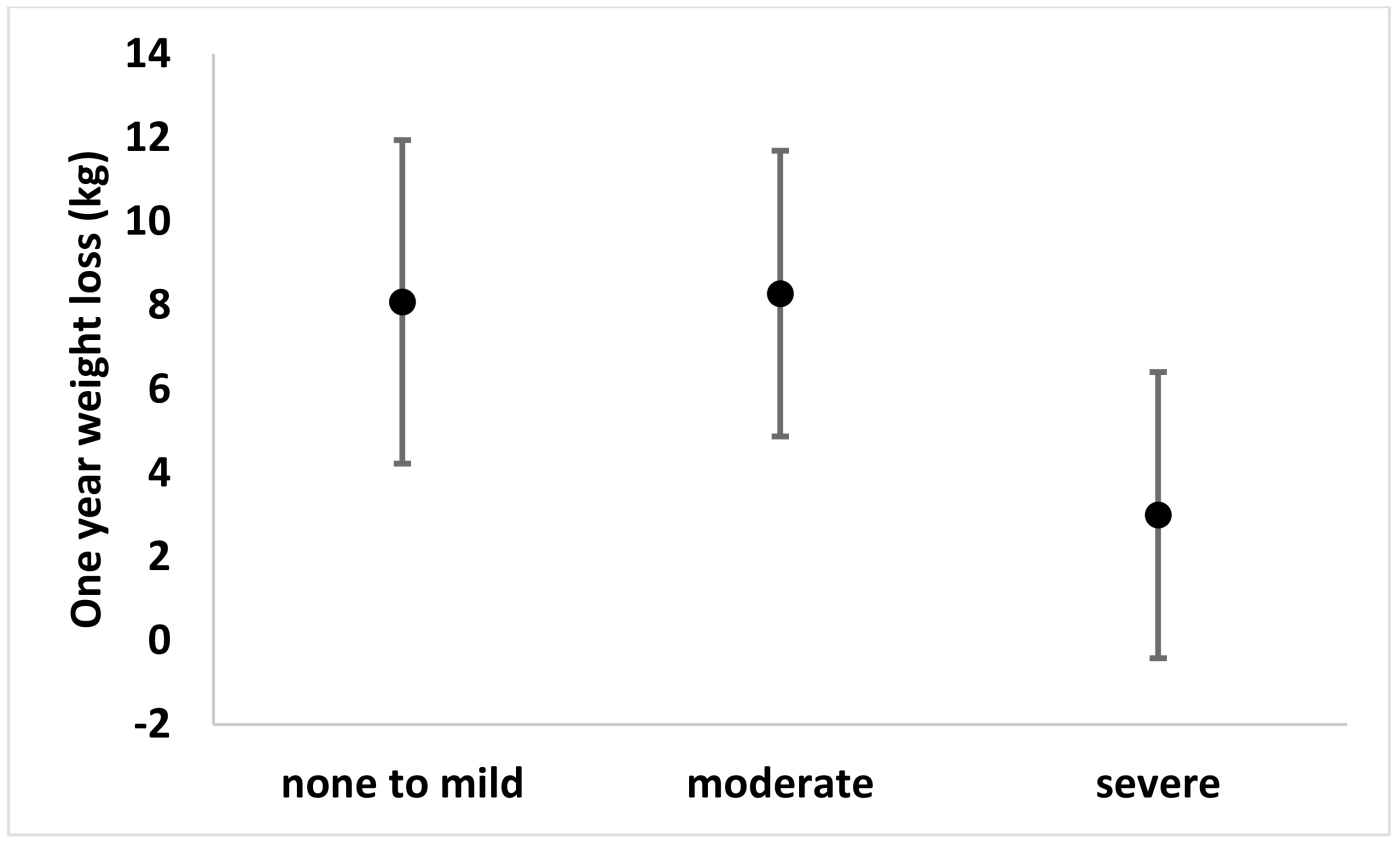
One-year weight loss separated by pain classification group.

Covariate-adjusted mean weight loss (95%CI) was similar for those with none-to-mild pain (8.1kg (4.2 to 12.0kg)) and moderate pain (8.3kg (4.9 to 11.7kg)), but was lower for the severe pain group (3.0kg (-0.4 to 6.4kg)) (Fig 1). There was evidence of an effect of baseline pain levels on weight loss after adjusting for all other covariates (p = 0.08). The mean difference (95%CI) in weight loss between the none-to-mild pain and the moderate pain groups was 0.2kg (-4.9 to 5.3, p = 0.94). The mean difference in weight loss for the severe pain group was 5.1kg (-0.6 to 10.7, p = 0.08) lower than the none-to-mild pain group and 5.3kg (0.4 to 10.2, p = 0.03) lower than the moderate pain group. The raw data used for the analysis can be found in supporting information (S1 Appendix).

## Discussion

This is the first study to directly investigate the effect of persistent pain on weight loss in patients undergoing specialist weight management within the NHS. More than 95% of the patients reported persistent pain at baseline with more than a third of patients reporting severe pain. Our findings indicate that patients with severe pain at baseline lost less weight at one-year follow-up when compared to those with none-to-mild pain or moderate pain. There was no difference between the none-to-mild pain and moderate pain groups.

The findings of our study are broadly in keeping with Masheb et al. [24] who found that people with severe pain lost significantly less weight -0.3kg (-1.8 to 1.2kg) than those with no pain -2.1kg (-2.7 to -1.4kg) or moderate pain -2.2kg (-3.5 to -0.09kg) and similar levels of weight loss between those with no pain and moderate pain [24]. The magnitude of the weight loss in our study was greater than that seen in Masheb et al. [24] who reported a group weight loss of 1.71% with 21.9% achieving weight loss of ≥5% in comparison to our study where there was a weight loss of 5.1% and 48% of patients achieved a weight loss of ≥5%. The difference in magnitude may be related to baseline obesity levels which were higher in the current study compared to that of Masheb et al. [24] (BMI = 46 vs. 36kg.m^-2^). Other reasons may be to do with differences in study methodology. Masheb et al. [24] was a reanalysis of an RCT investigating the effects of a weight loss intervention compared to a control in veterans, predominantly middle-aged males (85%), while our data was from patients receiving their usual care, predominantly females (70%). The differences may also have been cultural/geographical between the US and the UK. The magnitude of the weight loss in our study is comparable to that seen in conservative weight loss programmes in other parts of the UK (-4.8kg and -4.6%) [25].

The clinical implication of our findings is that severe pain levels may be a considerable barrier to weight loss in those referred to specialist weight management services in the NHS by a magnitude of 5kg. Given the high prevalence levels of persistent pain in obese populations, especially in those with more severe obesity [14], the reach and significance of pain as a barrier to weight loss may be considerable. As such, these findings support previous calls for better integration between weight management and pain management services [12,18]. Additional support may be warranted for patients with severe pain. Given that pain and its associated functional impairments are at least partly modifiable, targeting pain as part of a weight management strategy could potentially enhance weight loss outcomes for those with co-existing obesity and severe pain. There are a small but growing number of trials investigating the effectiveness of combined pain and weight management interventions in obese patients [34,35]. Our findings emphasise the merit of this work and suggest that such interventions may be best targeted at those with more severe pain.

This study has a number of limitations. This is a retrospective observational study, thus no claims of cause and effect can be made. While pain was measured using a valid and reliable questionnaire, pain characteristics such as location, duration and type of pain were not recorded. Thus, their potential role in weight loss was not explored. Whilst a number of important co-variates were adjusted for within the statistical model, some potentially important co-variates such as diet were not included. During the time period in which this data was collected, 837 patients were discharged from the specialist weight management service. Thus, data was only available for 19% of the patients at this clinic. As such, this sample may not be representative of patients attending NHS weight management services, reducing the generalisability of our findings. Additionally, the sample is small, which increases the risk of a type II error. However, the strength of this work is the use of a well-validated measure of pain and clinically established published cut-off values for none-to-mild, moderate and severe pain. Data on the location of the pain would have been useful contextual information but previous work suggests that it may be of limited relevance [24].

## Conclusion

In conclusion, patients with severe pain at the point of entry to an NHS specialist weight management service appear to lose less weight at one-year follow-up compared to those with none-to-mild or moderate pain. The magnitude of the difference is likely to be clinically relevant and highlights the potential gains in weight loss that might be achieved by addressing concomitant persistent pain in weight management services. There was no difference in weight loss between those who reported none-to-mild pain and moderate pain. These findings broadly support earlier findings in a sample of, predominantly male, US veterans [24], thus suggesting they are applicable to the NHS, which comprises of a high proportion of female patients. Future studies need to be conducted to more firmly establish the generalisability of these findings into the wider NHS setting, including applicability in non-specialised community weight management setting, which include patients with less severe forms of obesity. Future work investigating the feasibility of incorporating some form of pain management into the weight management setting is also be warranted.

## Supporting information

S1 Appendix Raw data used for the fully adjusted statistical analysis. (XLSX)
